# Cases, Chiefly of Accidental and Scrophulous Sores, Cured by the Internal Use of Cantharides

**Published:** 1808-10

**Authors:** John Roberton

**Affiliations:** Surgeon, Edinburgh


### Medical and Physical Journal.

VOL. XX.] OCTOBER 1808. [NO. 116.

*Printed for R. PHILLIPS, by W. Thorne, Red Lion Court, Fleet Street, London*

### CASES, CHIEFLY OF ACCIDENTAL AND SCROPHULOUS SORES, CURED BY THE INTERNAL USE OF CANTHARIDES.

By JOHN ROBERTON, Surgeon, EDINBURGH.

IN every period of the history of Surgery, the most successful plans adopted in the treatment of ulcers, seem all to have acted on the same principle; and in, whatever manner these various applications have been made, the substances employed have uniformly been of a stimulating quality. These have almost always produced escarotic effects, and been applied externally. Of late years, it is well known that the adhesive straps and roller have been successfully employed; and I may mention, that it is alone by stimulating the parts to which they are applied, that these can produce their beneficial effects.

The natural transition from producing and supporting such a state of action by any kind of external means, to that of effecting the same purpose by the external use of such medicines as cantharides, must be evident to every one who knows any thing of the extensively stimulating powers of that drug.

It is upon this principle that all other stimuli, applied internally to sores, &c. produce similar effects, though I have found, so far as my experience goes, none of them, in their operation, so permanently effectual as that medicine. I avoided mentioning this fact at an earlier period, because I wished to mature my observations on the subject. An individual, however, to whom I personally, among several other observations, probably of some importance, mentioned this one, has, *since the publication of my work on cantharides,* in which the principle was more than merely inculcated, thought proper, consistently with the the freedoms he has taken with every other author, to state it without any acknowledgement to me; although he knew

I intended to publish on the subject at a subsequent period. The Critical Review, however, for March 1808, and the Edinburgh Medical and Surgical Journal, published on the 1st of July last; indeed, every Review of his book, proves that I am not a solitary sufferer from the plagiarisms of the same person.

One of the misfortunes of such characters is, that, when they wish to plunder the labours of others, they know not really what use to make of them. Anxious to apply the same principle to digitalis, and thus have an opportunity of claiming it as his own, the same person publicly asserted the efficiency of that medicine in similar cases; and although he had no experience on the subject, dared to adduce, in proof of it, one of his cases, treated at Glasgow, and even presumed to call, as evidence to the truth of his assertions, the respectable Doctors Jeffery and Cleghorn of that city. The first of these gentlemen accordingly thought it his duty publicly to state the falsehood of these assertions, and to observe, that, if the other parts of the work were as little to be relied upon, it deserved to be committed to the flames. This, therefore, is a specimen of the errors that arise from attempts to claim principles of which the plagiarist knows not the nature.

Not satisfied with claiming principles of which he himself was ignorant, the same person has even persuaded a young man, just commencing the study of medicine, and of whom he has contrived to make a tool, but whose name I wish not to mention unless I am compelled, to claim the mere notion of applying cantharides to the cure of glanders in the horse. Although I first suggested this notion, I by no means stated it as a discovery, because experiments did not fully warrant me to do so. But, it appears, that arrogance and presumption are in the inverse ratio of knowledge.

I know that objections, from want of success, have been started to the treatment of several diseases by the cantharides. Such opposition I expected to meet with whenever I should make known the success of my practice with this substance.

It is a melancholy but well established fact in our profession, that the violence of opposition, which any improvement, either in surgery or medicine, meets with, is too often exactly in proportion to its importance in the removal of disease. While with the greatest facility, any mysterious agent which can be proposed for the recovery of health is, without scruple, admitted into universal use, that that which, to a demonstration, can be proved of the greatest benefit to the human race, is, when used, employed with fear and trembling, and seldom before a century or two elapse, so universally or to such extent as alone can insure all the utility of which it is capable.

To those who without prejudice, or a wish to condemn, give this substance a fair trial in such complaints as may indicate its use, and who really have been unsuccessful, I have only to observe, that I can very easily account for the failure of many medical practitioners in the treatment of diseases by cantharides. It was not till after many years experience, with the closest attention I could bestow to its operation, that I became at all successful in the removal of complaints with that medicine; and, even then I failed in the removal of some cases, which, with greater experience in the use of medicine, I have since completely effected. It will undoubtedly be with them, as it was with myself; but by cautiously persevering in its use, they will be sensible of its importance.

### CASE I.

A stout, and apparently healthy man, aged 55, about a year ago, had the middle of the sole of his foot completely perforated by a large nail. The nail was withdrawn, and the wound in a few weeks healed; but still some degree of swelling of that foot, especially towards evening, troubled him.

About four months ago, the ankle joint became somewhat more stiff than usual, and several small pustules broke out around the outer ankle, which soon formed very painful sores. In two weeks more the œdematous swelling had reached nearly to the knee, and several inches of the skin around the ankle joint had become of a dark red colour.

He now, (Nov. 3, 1807,) became alarmed at his situation, came to Edinburgh and put himself under my care. The only application he had made to his sores, was simple ointment with the constant use of a tight stocking, from which he had derived no benefit. I therefore prescribed tinct. canth. ℥ß. aq. font. ℥vi. M. a table spoonful of which was to be taken thrice a day, and the leg also to be rolled from the toe to the knee with a bandage of cotton cloth.—This medicine, in sufficient doses to produce slight yet constant pain in voiding urine, with the application of the bandage, completely cured him in a month. I however advised him, on going into the country, to continue the application of the bandage for several months.

Feb. 1808. Having about a fortnight ago fatigued himself by carrying heavy burthens almost for a whole day, he was seized with pain in the formerly affected leg, and it has now become œdematous, acquired a livid colour, extending about two thirds up the leg, and discharging a thin watery fluid from its whole surface. I ordered him to recommence the canth. as formerly directed, and again to apply the roller.

March 1st. The discharge from the diseased surface is thick and yellow, while the œdema and livid appearance are gradually disappearing.

He having occasion to go to London, and being now pretty well acquainted with the nature of the medicine, I have desired him to continue it in absence, and to write to me if any adverse symptom occurs.

May, 20th. This patient waited on me to day, to inform me of his leg having perfectly recovered. I have nevertheless desired him to avoid all fatigue for several months, and still to wear a roller.

July, 1808. This patient has, for two or three weeks laid aside the roller, and has had no return of his complaint.

### CASE II.

Nov. 6, 1806.—A carpenter, aged 29 years, about twelve years ago, cut his left leg with an adze. The sore has, since that time, been frequently skinned over, but it always, in a short time after, broke out again. He has applied to several Medical Gentlemen for relief, and, after every external application, from the mildest to the most corrosive, has been used without effect, he has for several years considered it as incurable, but while it permitted him to follow his ordinary occupation, he has been contented.

This patient has one sore near the middle of the tibia, about 2 ½ inches in diameter, with thick irregular edges, discharging thin brownish matter in considerable quantity, and several other sores about the size of a sixpence surrounding it; but these have only troubled him a year, and they are neither deep nor have they thick edges, although they discharge matter equally unhealthy with the large sore. The leg and foot are swelled, and about two thirds of its extent is of a livid colour. I have therefore prescribed tinct. canth. ℥i; aq. font. ℥vi. M.; a table-spoonful of which was to be taken four times a day.

18th. This patient has continued to use the cantharides in in sufficient doses to produce slight difficulty in voiding urine. The discharge has become uncommonly plentiful, is of tolerably good consistence, attended with stiffness and soreness in the ulcerated parts.

30th. He has continued to take the cantharides, and now the smaller sores seem nearly healed, but there is no alteration in the appearance of the large one. The discharge from it to day, and three days past, has been alarmingly great. I even believed it impossible for such a discharge to proceed from an ulcer of these dimensions, and suspecting sinus, I minutely examined the whole of its surface with a probe, but could discover none; the cantharides were therefore continued.

Dec. 7th. All the smaller sores were completely healed, but there was no alteration of the larger one. This day he became impatient, and took a very large dose of cantharides. About an hour afterwards he became sick and vomited, and during the night voided brown bloody matter along with his urine. He informed me of this circumstance, and I advised him to take a smart cathartic and use warm applications above the pubes. The cathartic was rejected by vomiting, and indeed, for two days, nothing would remain on his stomach, and it was not till after he had suffered the most excruciating pain, that these symptoms began to abate. The sore has now become so irritable that he cannot even allow the weight of the common dressing to press on it.

17th. The above symptoms have disappeared, but the sore does not seem to have been much affected by what has been done.

20th. This day I began to use a solution of the muriate of mercury as a wash, and desired him to recommence the cantharides, none of which he had taken since the 7th.

29th. The sore now began to have a very favourable appearance, the whole surface looking florid, the edges becoming thinner, and its diameter not being so great as formerly. The cantharides and solution were therefore continued.

Jan. 20, 1807. The sore now evidently contracted in size. The solution was applied where the edges seemed not disposed to heal, and this produced the most beneficial effects. The cantharides were still continued.

March 25th. The sore remained nearly in the same state since last report, although he had taken the cantharides in sufficient doses to affect his urinary organs.

April 6th. The sore now assumed a black unhealthy appearance, pearance, and became thicker in the edges. He had continued to take his medicines regularly, and in every respect to obey my prescription. My patient could only account for this change of appearance from his having about three days ago exposed himself to very severe fatigue for a whole day; since which time the sore had gradually degenerated.

May 20th. Although he had taken the cantharides regularly, and in sufficient doses since last report, the sore had still continued to degenerate, and it was now larger than I ever saw it. I certainly would now have omitted the use of the cantharides for the present, had it given him any uneasiness at the stomach, or seemed to affect his pulse in an unfavorable way, but as all went on well, I desired him to continue.

26th. The sore has again assumed a more healthy appearance, and discharges thicker matter.

August, 1807. He had regularly continued the cantharides since last report, and his leg was now perfectly well, and he himself able to work at his trade.

July, 1808. This patient still remained perfectly well.

### CASE III.

A woman, aged 35, of a robust habit of body, was, about 18 years ago, affected with Erysipelas in her right leg, which occupied the whole space from the knee to the point of her toes. Leeches were applied over the inner ankle, and the wounds made by them degenerated into foul ulcers. These, however, soon healed, and the redness entirely disappeared from the limb. She still complained of weakness in that leg, and being a kitchen servant, and obliged to fatigue herself considerably, she was often before night scarcely capable of supporting her weight upon it. Two years after this, she twisted the ankle joint of this leg; and that part of the leg which had been formerly ulcerated, had the skin rubbed off it, and an astonishing quantity of blood was discharged by the opening. This wound, however, was soon healed, but the leg was always in a greater or less degree swelled for eight years afterward. About that time she had another attack of Erysipelas in the same leg, which she attributed to her living in a damp house at that time. This disappeared in about a fortnight, and the ulcerated part formerly mentioned, broke out again. It was again healed by the application of ointment, but still the swelling continued, particularly toward the evening.

Eighteen

Eighteen months ago, after undergoing considerable fatigue, the swelling in her leg increased to an amazing extent, the ulcerations again commenced, and varicose veins appeared in different parts of it. She became greatly alarmed at this appearance, and applied for assistance at the Royal Infirmary of this place. There, adhesive straps were applied to the ulcerated parts, and bandages of cotton cloth were used. She was dismissed from the Infirmary perfectly cured in less than a month.

In October last she was again exposed to dampness and fatigue, and the swellings and ulcerations on the same place again broke out. She again applied to the Infirmary, where the same practice was followed as formerly mentioned. From this she derived great benefit, and was a second time dismissed cured. A few days after she left the House, the ulcerations became much worse, and the swelling increased. This last she kept under by bandaging, but the ulcerations continued to extend; and Feb. 1, 1808, when she applied to me and gave me the above account of her disease, her leg, from about a hand breadth below the knee to the upper part of the foot, was of a livid colour. The varicose veins still existed all over the diseased surface of the leg, and there were two ulcers above the inner maleolus, the smallest being about an inch in diameter, and the other of an irregular form in appearance, and about six or eight times the size of the other; both of them discharging thin matter. I prescribed tinct. cantharid. ℥iß. twenty drops to be taken thrice a day in a glass of water, and I desired her to apply a tight roller of cotton cloth to the diseased leg from the point of the toes to her knee.

10th. The sores look very clean, but she has experienced no effect on the urinary organs from the use of the cantharides, which are to be continued.

13th. Slight pain now occurred in voiding urine, and, in different parts of the largest sores, granulations are distinctly seen shooting across it, but not the smallest diminution in its size has taken place. The œdema of the leg has decreased in a surprising degree, but the varicose veins and livid appearance remain as formerly; I therefore ordered the cantharides, &c. to be continued.

28th. The sores were now healing gradually, and the general appearance of the leg was much improved. She experienced no weakness in it, being able to use it without any inconvenience from the greatest exertion. She has frequently felt pain in voiding urine, but this was only in a slight degree. The cantharides, &c. were, therefore, continued. There was, till the 20th of April following, little variation in the daily reports, and the wounded surface being at that time quite healed, she went into the country.

### CASE IV.

A man, aged 35, about five years ago, fatigued himself by walking a great deal, which was immediately followed by a swelling in both legs; the skin also broke in several places; and, in the course of a few weeks there was a constant discharge from them of a thin watery fluid, attended with acute pain from the knees to the ankles. Mercury was prescribed, which weakened him very much, but did not in the least relieve him of his complaint. After he gave over the use of this medicine, the swelling began to abate, but the eruption, with the discharge, continued to extend to his thighs; and, in a few months, it covered his whole body, and even his arms to the points of his fingers. All the diseased surface discharged thin matter, similar to what was at first discharged from his legs, attended with some pain, but his legs were always more so than any other part of his body. He then came from the country into the Infirmary of this city, but derived no benefit from the prescriptions he received there. In consequence of this disease, he had lived in great misery for four years past, during which time he had used almost every external application that whim or superstition could suggest, but derived no benefit from them. Of late he had used ung. citrin. which he thought kept the parts soft, but did no other good. I desired him to lay aside all external applications, and I prescribed on the 1st of Nov. 1806, tinct. canth. ℥ß. aq. font. ℥vi. M. A table spoonful to be taken four times a-day.

4th He repeated the mixture with ʒj. of cantharides. That evening he felt slight pain in voiding urine, and a very disagreeable sensation all over his body.

7th. He repeated the mixture, and his symptoms were as formerly.

10th. Itching all over the body had become very troublesome to him.

16th. It was now with the greatest difficulty I could prevail on my patient to continue the tincture, on account of the very disagreeable sensations it produced all over the body, particularly as the eruption seemed to him worse than before he began the use of it,

20th. This

20th. This morning he felt less of that soreness and disagreeable sensation than formerly, particularly about the lower parts of his legs where the disease first began. I still ordered the cantharides to be continued.

24th. The itching does not now trouble him in his legs, and it is beginning to decrease along the thighs. The cantharides still to be continued.

Dec. 14. His legs were now perfectly well, but the other parts of his body continued as last reported. For two weeks past, he had taken about ℥j of the tincture daily, and I desired him to continue it.

28th. I now diminished the dose of the cantharides to ʒjv. per diem, on account of its violent effects on the urinary organs. The itching was not so general as formerly, but, where felt, it was equally severe as before the use of the cantharides. The discharge from the eruption was now entirely gone, and his legs were as well as ever. He had all along observed that the affection of his urinary organs was uncommonly severe, a few hours after he used any kind of spirituous liquors. I ordered ʒjv. of the cantharides to be taken daily.

Jan. 6. The eruption seemed now to be disappearing very rapidly from every part of his body, and he was stouter and in better health than he had been since the commencement of his complaint. I desired ʒiij. of the cantharides to be continued per diem.

16th. Two days ago, a spot above the right eye became inflamed and painful; spread in every direction; and, in two days the right eye was much inflamed, and likewise the upper part of the right cheek. This diseased surface discharged a thin watery matter. The part was bathed with spt. vin. and a few drops of tinct. thebaic were dropped into the eye. In a few days this affection was quite removed; the eruption over the body was now almost completely well; I however ordered the cantharides to be continued.

Feb. 1st. The eruption on various parts of the body was equally severe as before the use of the cantharides. I therefore desired him to continue the cantharides, and to bathe the affected parts with spt. vini.

20th. The eruption had entirely disappeared, and he was, in every respect, perfectly well. I nevertheless still ordered the cantharides to be continued in small doses.

April 2d. The eruption again broke out in three different parts of his body. He had taken no cantharides for about ten days; I desired him to recommence it in small doses.

May

May 1. This patient was now completely recovered, but I desired him to continue the cantharides a few weeks, and to inform me if his complaint returned.

July 10th, 1808. I was this day informed that this patient still continued perfectly well, and free from his complaint.

### CASE V.

Dec. 24th, 1806. A Gentleman aged 35, lame, and of a weakly habit of body, applied to me in September last, with a small tumour, of a livid colour, situated about three inches toward the right side of the thyroid cartilage, which evidently contained fluid matter; but he would not submit to have it opened, and he went into the country. This day he returned, and informed me, that the tumour never had increased beyond the size of a small walnut, and had never given him pain. It had broke and discharged thin matter about the beginning of the present month, and a surgeon, in that part of the country, advised him to apply a poultice for a few days, and afterwards to dress the external sore with ung. basilic. He continued however to observe these instructions, without benefit, till he came to Edinburgh. On examination, I found two small openings discharging thin acrid matter in great abundance. I introduced a probe, which passed easily forward over the anterior surface of the trachea, forming a cavity of considerable extent. By dissecting off the integuments, I laid the sinus completely open, and thus an ill-conditioned sore was formed, extending three inches across the fore part of the neck, and one and a half from above downward. I prescribed tinct. cantharid. ℥j. aq. font. ℥vj. M. A small wine glass-full to be taken thrice a day. This mixture was repeated on the 27th, when he informed me, that he felt a more frequent inclination to void urine than usual, and when this operation was over, slight pains troubled him for some time about the neck of the bladder. The cantharides were therefore continued.

29th. He now complained of stiffness in the sore, which appeared somewhat inflamed, but the nature of the discharge was unaltered, and the pain in voiding urine as formerly.

Jan. 6th. Since last report, he has kept the urinary organs slightly affected by the use of the cantharides, and now the discharge is puriform, and the sore is very painful. I this day removed several portions of loose skin about two lines in breadth round the edge of the sore. He complained plained of great pain during this operation, and the wounded surface bled freely. The cantharides were still continued.

12th. Granulations, very exuberant and somewhat elevated above the sound parts, now appeared; I therefore ordered very moderate pressure to be applied, by means of a pad of linen, and the cantharides to be continued.

18th. In some parts of the sore, granulations elevated above the sound parts continue, in others small ill-looking sinuses are forming, which discharge thin acrid matter, so that at present two states of action, very different in their nature, are going on in the sore. As the sinuses form, I lay them open, and bathe the parts with spt. vini. and apply slight pressure to the other parts. The cantharides were still continued.

Mar. 1. For the last six weeks the cantharides have been continued, several sinuses have been formed, which I have laid open, and other parts of the sore have healed. His urinary organs were now so easily affected, that the smallest dose of the cantharides pains him severely for several hours. I therefore desired him to omit the cantharides, and use as a substitute about a pint of wine per diem, and the sore to be washed with spt. vini. This day he went into the country.

April 3d. He returned from the country; the sores did not look so healthy as formerly, and he expressed a wish to recommence the tinct. cantharid. as the wine produced very uneasy sensations in his stomach. I desired the cantharides to be taken in small doses.

27th. The sore had healed rapidly since last report, and his general health was much improved. The cantharides were continued.

May 20th. The sore was now completely healed, I therefore desired him to omit the cantharides, and anoint the tender surface with axung.

June 1. Two or three small pimples, containing thin matter, had appeared on the recently healed surface; I therefore desired him to recommence the cantharides in small doses, and the parts to be washed with spt. vini.

8th. The surface now was completely skinned, and a beautiful plexus of tender blood vessels were observable all over the formerly diseased surface. I desired him still to continue the cantharides for a week.

August 13th. This patient continues perfectly well, and the livid appearance, which formerly occupied a considerable space, has now nearly assumed a healthy aspect.

August,

August, 1808. He continues quite free from his complaint, and the scar is scarcely perceptible.

### CASE VI.

June 1, 1807. A boy, aged 11, small in stature, but of a healthy appearance, had till about 18 months ago, enjoyed good health, when, without his parents or himself being able to assign any cause for it, two phlegmonous swellings, about the size of a walnut, made their appearance on the outside of and rather below the elbow joint. They broke, and the discharge, which was small in quantity, was of a thin consistence. He applied to the Royal Infirmary of this place, where he was ordered to dress the sores with the calamine cerate of the shops. He continued this application three weeks without seeming to derive any benefit from its use, when another phlegmon made its appearance three inches farther up the arm than the former, which likewise broke, and discharged matter of a similar consistence. The same kind of dressings which were employed in the other sores, were likewise applied to this.

About the beginning of September following, he received a kick from a horse on the fore part of the ankle joint; this was followed by considerable swelling of the under part of his leg, which in a few days terminated in a phlegmon near the ankle joint. This broke and discharged matter similar to what was discharged from his arm, and the motion of the joint was considerably impeded. He was desired to apply poultices around the ankle, and over the sore, but from them he derived no benefit, although he continued their use for several weeks. He now began to suffer considerably in his general health, and another sore of a similar nature to the last, rather more toward the outside of the leg, appeared. Sea bathing, with a great variety of dressings, were employed, but from these he derived no benefit.

He applied to me the 1st of June, 1807. His general appearance at that time indicated considerable debility. The above mentioned sores looked remarkably unhealthy; those on the leg, in particular, had thick callous edges, and the ankle joint was considerably enlarged and stiff. He walked on the point of his toes, and kept his knee joint constantly bent, because he could not stretch it without occasioning great pain in his ankle joint and in the sores. The flexors of his fore arm were so much contracted as to cause the fore arm to form a right angle with the humerus.

As

As none of the glands of any part of his body seemed preternaturally enlarged, and as the sores, which were evidently of a scrophulous nature, seemed in their general character similar to what I had treated successfully with the cantharides, I prescribed tinct. cantharid. ℥ij. aq. font. ℥vj. M. A table spoonful to be taken thrice a day, and all the sores to be dressed with simple ointment.

June 2. Another sore, nearly of the same character, broke out on the same arm, a little below the former. From this, however, was discharged a great quantity of bloody mixed matter.

4th. A sort of bag, seemingly containing watery matter, appeared to rise from one of the sores near the ankle joint, which broke this day, and discharged a coasiderable quantity of watery matter. He now felt much pain in voiding urine, which he was inclined to do very frequently, and a sort of darting pain in the situation of the kidneys. I therefore desired the cantharides to be taken in diminished doses.

8th. For the last three days he has taken only three tea spoonfuls of his mixture daily, on account of the great pain he felt in voiding urine. The sores were now more painful, and the discharge from them more thick in consistence, and whiter than he had ever observed it; the swelling too on the foot and ankle joint had abated considerably.

13th. The sores had all become very painful, particularly the large one on the front of the foot, and all of them discharged healthy pus. He continued to pass his water very freely; the cantharides were therefore continued.

16th. There had been very little discharge from the sores on his arm for several days, but those on his legs continued to discharge freely, and the swelling on the foot and ankle joint had completely disappeared. He had felt very little pain from his medicine, I therefore desired him to take an additional tea spoonful per diem.

20th. The pain in voiding urine again became very troublesome, also pain extending along the whole leg, with a considerable degree of swelling. The sores, however, which are very painful look well, and the discharge from them is very good. The cantharides were discontinued for a few days.

21st. A phlegmonous swelling had appeared on the forepart of the leg since yesterday, to which I ordered a poultice to be applied. The general pain all over the leg had abatabated, but the urinary organs were still considerably affectedb y the cantharides.

25th. Poultices were constantly applied to the tumour, till this day, when distinct fluctuation of matter was felt in it, and it was laid open. There was discharged from it a great quantity of blackish fœtid matter. After this a poultice was applied to the wound; I desired him to recommence the cantharides in small doses.

27th. The last mentioned wound seems very deep and black, I therefore desired spt. vini. to be injected into it twice a day, and cantharides to be continued.

July 1st. All the sores now looked well, and the swelling mentioned on the 20th ult. had disappeared completely. The canth. were still continued.

6th. A small tumour had appeared within two inches of the phlegmon last mentioned, which broke during the night, and discharged a small quantity of bloody matter. The cantharides were now continued in increased doses.

14th. The sores all looked very healthy, and the discharge from them was good, and in moderate quantity. I desired him to dress them with simple ointment. He continued to void urine freely, and the cantharides were therefore continued.

24th. The sores on the foot were evidently beginning to heal, and those on the arm to look clean and healthy. The cantharides were still continued.

28th. All the sores were now healing very fast, some of the smaller ones were indeed completely skinned over. His general health too was greatly improved, and he took his victuals remarkably well.

Sept. 1st. Adhesive straps and roller were applied to the sores, both of arm and leg, and the cantharides were continued.

12th. Almost all the sores had healed, I however ordered the adhesive straps and roller to be still applied.

Oct. 3d. All the sores had completely healed, I desired nevertheless the roller to be continued, and the cantharides also in small doses for a week or two.

16th. A phlegmonous swelling appeared on the anterior part of the ankle joint, to which warm poultices were applied, and on the 22d it broke, and discharged about a table-spoonful of matter of a thin consistence. The cantharides were continued.

Nov. 1st. The sore was now completely healed, I however desired the cantharides to be continued in small doses, and the roller to be constantly applied.

25th. This

25th. This patient continued in perfect health, and all the sores were healed. The cantharides and roller were still continued.

Dec. 20th. He refused to take any more of that medicine.

Jan. 2d, 1808. A small tumour with an inflamed base appeared on the posterior part of the wrist of the arm, which was formerly affected. I desired poultices to be applied to it, and the recommencement of the cantharides.

20th. The tumour was opened, and discharged matter of a better consistence than any of the others, which were formerly opened. The edges of the sore were hard, thick, and inflamed to some extent round its base. I still desired poultices to be applied, the cantharides to be continued, and spt. vini. to be daily injected into the sore.

No new sores afterwards appeared, and, by a continuance of this plan, the above one was completely healed in less than three weeeks. I however desired him to continue the cantharides in small doses for two or three weeks longer.

August, 1808. This patient continues perfectly well, and has, since the removal of his complaints, become much stronger than ever he recollects having been.

This case was principally attended to by Mr. John Lizars, apprentice to Mr. John Bell.

### CASE VII.

A wood-cutter, aged 24, stout made, was, about seven years ago, affected with scrofulous swellings in several parts of his body. Only those however of his neck suppurated and broke. He applied to me the 22d of April 1806; and as it may be of some importance to convey to the reader an idea of the general appearance of his disease, I shall attempt to describe it.

On the right side of his head and neck there is one sore, between the sterno-cleido-mastoideus and masseter, extending from the lobule of the ear to the angle of the inferior jaw, and discharging matter by two orifices. A second sore, commencing about an inch from the former, is situated on the check, its upper edge being opposite the termination of the parotid duct, itself extending downwards, uniting with another sore under the maxilla, and stretching along in that direction. These discharge matter by four orifices. Another sore is situated between the trapezeus, and sterno-cleido mastoideus, and extends across the inferior extremity of the platysma myoideus to the sterno-hyoideus. A

very very large sore extended from the inferior edge of the thyroid gland, over the sternum to the insertion of the second pair of ribs, and was covered with scabs on this side. There is also a sore running across under the chin, which unite the sores on opposite sides.

On the left side the sores were much more extensive than on the opposite side, and not so capable of the same distinct description. They seemed however to form three lines, one commencing behind the mastoid process, extending downwards till near the acromion. The second from behind the lobule of the ear to the anterior half of the clavicle; and the third from the zygomatic process of the temporal bone, over the masseter and upper part of the platysma myoides, joined as above described, with the sores of the opposite side. The matter was discharged here by numerous orifices, and approached somewhat to the appearance of laudable pus. His neck is in general very much enlarged, it being at least equal in circumference to any part of his head.

The axillary glands are but slightly enlarged, but all the glands in both groins, along the course of Poupart's ligament, and extending down upon the inside of the thigh, were universally enlarged.

The disease began by the sore above the trachea, which broke about five years ago, after having been in a diseased state full two years; and all the other parts mentioned about the neck becoming also affected, broke three years ago. The whole neck and face continued more or less swelled. The general health however continued all the while pretty good, except from to time severe pains in the bowels affected him, which were eased by the discharge of flatus downward. He does not recollect if he was at these times costive.

When he applied to me, he complained of loss of appetite and sickness, in consequence of which he had been unable to work at his trade for several months. From the general tendency to glandular swellings in this case, I conceived it more prudent to employ the solution of the muriate of lime than the cantharides, as in several cases of very large glandular swellings, I found this medicine, when used in large doses from ʒiv. to ℥j. per diem of the greatest service. I know that this valuable medicine, like many others, has been nearly consigned to oblivion, from an idea propagated by men thought to be eminent in their profession, that it is possessed of no useful quality; but this is not very uncommon in our profession, for, unassisted by reasoning of any any kind, one medicine after another has been applauded, has retained its popularity for a length of time, and, as might be expected, has at last been completely neglected. Not that the medicine wanted power, but that its employers wanted judgment. I prescribed this medicine to be taken at first in doses of ℥ß per diem, to be gradually increased. I at the same time prescribed as tonics Peruvian bark and rub. ferri, to restore the appetite and promote digestion, desiring the ulcerated parts to be bathed with warm sea water, and dressed with simple ointment.

May 30. I prescribed the following solution to wash the sores, sulph. cupri ʒß. aq. font. ℥vi. M. f. solut.; muriate of lime continued. This day there is a tumour, which began two days ago, situated over the jaw, and directly under the dens caninus. It is now as large as a walnut, and completely filled with matter.

June 7th. This tumour has shrunk and nearly disappeared without any external opening, and the fluctuation of matter is scarcely to be felt in it. The sores have a more healthy appearance than he says they have had since the commencement of the disease. No new swellings or sores have broke out since he began to take the muriate, and he is now able to work at his trade, is free from sickness, and takes his victuals well.

29th. I repeated the mixture as formerly, the patient still continuing to get better.

July 11th. The swelling of the glands were greatly removed, but this day the ulceration was worse, and very universally spread all over his neck, for the most part in small distinct pustules, discharging thin white matter. I then determined to give up the use of the muriate, and try the cantharides, which I had hitherto declined doing on account of the swelling of the glands; but, as he was obliged to return to the country before he could arrange matters to stay in town during its use, he therefore did not begin to take it till this day, August 9th.

The sores still discharging unhealthy matter, and there is a tumour the size of a walnut almost above the trachea, in which matter evidently fluctuates. I prescribed tinct. canth. ℥ß. aq. font. ℥vi. M. A table spoonful to be taken four times a day.

He continued to use this mixture without any perceptible effect, till the evening of the 12th. While in bed he felt intolerable itchiness all over his body, and on examining his skin, he discovered blotches of the size of a shilling completely covering him, and appearing as if he had been stung by nettles.

13th. He informed me of the above circumstance on the evening of this day, when the blotches had almost all disappeared, and he felt a kind of soreness on several of the parts where they had been. He had taken none of the mixture since last night, being terrified lest the blotched appearance of his skin should return. The discharge from the sores was of a thin watery appearance, except in one small spot under the right ear, from which thick white matter was discharged, in greater quantity than was poured out by any other of the sores of a similar size. No effect had been produced on the urinary organs. The tumour mentioned on the 9th broke this morning, and discharged thin matter. I have ordered him to poultice it, to recommence the cantharides and repeat the mixture with ℥vj. of the tincture.

15th. No effect was produced by the cantharides on the urinary organs, nor had the blothches troubled him again. Several of the sores discharge thin, others thick, white matter; formerly they all discharged thin ill conditioned matter: the discharge is likewise increased in quantity, and, such is the nature of the sores, that if pressure be made on part of his neck, formerly described to be in a diseased state, matter of different colours and consistence can be squeezed out as if from a sponge; yet a probe passes but a very small way into either of the openings. I desi-ed four table-spoons full of the last mixture to be taken per diem, and dressings of simple ointment to be used.

19th. The probe went much easier into the openings than yesterday. In one situated over the parotid gland, I could introduce a probe nearly an inch all round, and there was discharged from it thin matter in considerable quantity. He was now very timorous, and would not submit to have it laid open. Several of the sores however were completely healed. Some still discharged thin, some thick matter, and there were several small papulæ with white tops on several parts of his neck, which never were seen before. No effect was produced on the urinary organs, I therefore desired the cantharides to be continued in increased doses.

20th. After he went to bed last night, there was some pain in his urinary organs, and difficulty in passing water, but these went off before morning. I still desired him to continue the cantharides.

22d. I now judged it necessary to lay open the sinus over over the parotid gland, but the patient would not submit to it; I therefore declined doing any more for him till he comes to have a more correct sense of his duty.

Sept. 1st. My patient returned to me this morning with a determination to submit to whatever measures I might think necessary for the removal of his complaint. The discharge from all the sores had become thin, and, from some of them, it was perfectly limpid. I found the formerly mentioned sinus over the parotid gland considerably enlarged in extent since the 22d, and I made an opening from the uppermost to the most depending part of it, in length 2 ½ inches. I opened another about 3 inches in length, in the direction of the sterno-cleido mastoideus. I likewise opened several smaller sinuses, extending two or three lines immediately under the integuments of the neck. I filled all of these with lint dipped in sp. vini. and ordered him to recommence the use of the tinct. cantharides as follows: ℞. Tinct. canth. ℥j. aq. font. ℥vi. M. A table spoonful four times a day.

3d. The discharge was now thicker and the sores looked a good deal inflamed, but no effect was produced on the urinary organs. He complained of stiffness and soreness when he attempted to move his neck. I therefore desired him to continue the cantharides.

4th. He repeated the mixture as formerly, but complained of no pain or difficulty in voiding urine. The sores now discharged good pus, and in moderate quantity. I ordered the cantharides to be continued.

6th. He felt some difficulty and pain in voiding urine, but it soon went off, and the cantharides were continued.

7th. I opened several small sinuses about two lines in depth. All the sores now discharge healthy pus, and appeared considerably inflamed. I ordered the mixture to be repeated, and to be taken in sufficient doses to keep up slight difficulty in voiding urine.

11th. Since I saw him on the 7th, a tumour about three inches in circumference, immediately above the angle of the jaw on the right side, has been formed. I opened this, and there was discharged from it healthy pus. He complained of stiffness and soreness in attempting to move his neck, and all the sores discharged healthy pus: several of them have even healed since last report. I desired the mixture to be repeated with ℥iß of the cantharides, and in increased doses, as he felt no effect on his urinary organs for two days.

15th. I

15th. I laid open a number of small sinuses on the right side of the neck, and one extending along the whole anterior edge of the sterno-cleido mastoideus. I ordered the cantharides to be continued.

17th. The discharge from the last opened sinus was now very profuse and healthy; the sores were of a florid healthy appearance; and he felt stiffness and pain on moving his neck. He has felt slight difficulty in voiding urine. I, however, ordered the cantharides to be continued.

20th. He had taken an ounce of the tincture per diem since the 15th, and had felt only slight difficulty in voiding urine. The sores on the right side of the neck were now healing very rapidly, and discharging a very moderate quantity of healthy pus. On the left side of the neck I found a very extensive sinus, somewhat similar to the one above described, but I declined laying it open till some of the sores on the other side should heal. The cantharides still continued.

27th. The extensive sore laid open on the 15th, was now nearly healed. But while some parts of the other sores are healing, other parts of them do not look so healthy. I desired them to be dressed with ung. nitrat. rub. The cantharides continued to produce slight effects on the urinary organs, the dose for some days past having been ℥iv. per diem.

Oct. 1st. The sores were healing very slowly; I therefore desired nourishing food to be taken, and the cantharides to be continued.

8th. An indolent tumour, the size of a pigeon's egg, which for some years past had existed in one of his axillæ, became painful, and I have ordered him to discontinue the cantharides, and apply to it at first leeches, and then cold solutions, while at the same time he took a smart cathartic. The sores still inclined to heal slowly.

16th The above application seemed to have very little effect in allaying the swelling, for it this morning broke, and discharged a great quantity of mixed matter. As there was no perceptible swelling of the gland, I desired him to recommence the cantharides, and to apply warm poultices to the axilla. The matter discharged from the sores in the neck had evidently become thinner within these few days, but, upon the whole, his general health is very good.

22d. Dry hard scabs now formed on several of the smaller sores, which, in one or two instances, I removed, and found found a collection of thin matter under them; I therefore in future shall endeavour to prevent their formation. The cantharides were still continued.

30th. I laid open the extensive sinus on the left side of the neck, and bathed it with sp. vini. The other sores continued to discharge healthy matter, and are healing slowly. I desired ʒij. of the cantharides to be continued per diem.

Nov. 10th. His neck, which before was much deformed, and greatly enlarged, had now nearly its natural shape and thickness, and, except the last opened sinus, all the most important sores had healed. There were however, several small pustules scattered over the formerly diseased surface, discharging healthy matter. I ordered ʒij. of the cantharides to be taken per diem, and gradually and slowly diminished.

Dec. 20th. Nothing now remains of the above disease, but a few pustules, which alternately heal, and are formed on different parts of the formerly diseased surface. I ordered the parts to be daily bathed with sp. vini. and ʒj. of the cantharides to be taken daily. He was now in as good health, and was as capable of working at his trade as ever he was before the disease affected him.

Feb. 12, 1807. As the sores had now been completely healed for two weeks, and only a sort of stiffness remained in the neck, I desired him to take no more of the cantharides, and when the weather permitted to use sea-bathing.

Aug. 1808. This patient remained perfectly free from his complaint, having had no eruption or sore on any part of his body for more than a year.

This patient is at present—a servant of Lord Hopetoun's.

---

The cases which I shall next month communicate to the Journal, will be those of affections of the generative organs of both sexes, generally treated by the same medicine. There may exist similar cases in the writings of medical authors, but I have not met with any such; and the plan of treatment which these indicate, has been in every instance successful.

